# Simulating the Structure of Magnetic Fluid Using Dissipative Particle Dynamics Method

**DOI:** 10.3390/ma18081697

**Published:** 2025-04-08

**Authors:** Xiaoxi Tian, Fanian Lai, Yu Ying

**Affiliations:** School of Electrical and Control Engineering, Shenyang Jianzhu University, Shenyang 110168, China

**Keywords:** magnetic fluid, dissipative particle dynamics method, chain-like structures

## Abstract

Magnetic fluids (MF), composed of ferromagnetic nanoparticles, surfactants, and a carrier liquid, exhibit tunable physical properties under external magnetic fields due to the formation of chain-like nanoparticle structures. Using dissipative particle dynamics (DPD), we simulate the structural evolution of these fluids and establish a computational model incorporating magnetic nanoparticles and solvent particles. Our simulations confirm qualitative agreement with the literature results, validating the chosen time integration methods. Through radial distribution function analysis, we further demonstrate how the mass of solvent molecules and magnetic interaction strength govern the fluid’s microstructure. This work provides insights into the design of magnetic fluids for applications such as targeted drug delivery, adaptive dampers, and advanced magneto-rheological devices.

## 1. Introduction

MF is a type of stable colloidal solution where magnetic nanoparticles are dispersed in a liquid carrier, aided by the presence of surfactants and the Brownian motion [1]. Since their invention, magnetic fluids have been widely applied in various research fields, such as biomedical engineering [2], environmental engineering [3,4], aerospace technology [5], mechanical engineering [6,7] and sensing technology [8,9,10,11]. Due to the physical properties of magnetic fluids themselves, particularly their relationship with optics and magnetic fluid structure, it is crucial to understand the properties of magnetic fluids at a microscopic level. This understanding can be achieved through modeling and analysis using simulation systems for magnetic fluids. Moreover, numerical simulation studies of the microstructure of magnetic fluids under the influence of an external magnetic field not only offer a more economical and environmentally friendly approach but also allow for the observation of changes that are difficult to capture in experiments, thus compensating for the limitations of experimental studies.

In recent years, the study of microstructure has attracted significant interest among scientific researchers. To enhance research efficiency, protect the environment, and reduce costs, numerous efforts have been devoted to investigating numerical methods for studying the structure of magnetic fluids. These methods include Molecular Dynamics (MD) [12], Monte Carlo (MC) [13], Lattice Boltzmann (LB) [14], Brownian Dynamics (BD) [15], and DPD [16]. In 2013, LV et al. laid the foundation for transmission simulation by using the Monte Carlo method to construct the microstructure of MF. Subsequently, they experimentally revealed the movement of magnetic particles in magnetic fluid films under different magnetic fields, demonstrating the feasibility of microstructure simulation and providing effective theoretical support for transmission simulation [17]. In 2016, Li et al. employed the DPD-based algorithm MVVA-MVVA to simulate the 3D microstructure of ferrofluids. They introduced the model of magnetic fluids and systematically studied the influences of magnetic particle interaction strength and volume fraction, yielding various aggregate structures that aligned well with those obtained using other numerical approaches, demonstrating the effectiveness of the DPD-based approach [18]. Moghadam et al. investigated a mini-magnetorheological damper using the DPD method for molecular modeling, with validation against experimental data achieved through a modified Bouc-Wen model [19]. In 2021, Xu et al. utilized a research method combining mathematical analysis and performance testing to expand the application scope of MR fluids. A micromechanical model considering volume fraction and magnetic induction effects on microstructure evolution is proposed for MR fluids with MWCNTs/GO coated CI particles. The shear yield stress test conducted on self-prepared MR fluids using an MCR302 rheometer is compared with theoretical values from the model to validate its effectiveness [20]. In the past 20 years, MD, MC, and LB methods were the main approaches in the simulation of structure of magnetic fluids. However, there is a very limited literature dedicated to the study of the structure of magnetic fluids using a DPD-based method, but the DPD method is a very effective mesoscopic simulation technology for simulating the magnetic fluid’s structure.

DPD is a mesoscale simulation method for studying complex fluids and soft matter dynamics, offering a robust framework to analyze solution systems and advance theoretical understanding in the field. In this work, the DPD method is employed to calculate the microstructure of magnetic fluids. In order to accurately reflect the real state, a two-dimensional simulation model was first established based on Fortran language, capturing various aggregate structures. After comparing different time integration algorithms, the Verlet algorithm was selected for model update calculations. Subsequently, the system studied the effects of the weight of carrier liquid molecules and the strength of magnetic particle interactions on the microstructure of magnetic fluid particles. This study provides valuable guidance for subsequent experimental research.

## 2. Methods and Frameworks

**Figure 1:**
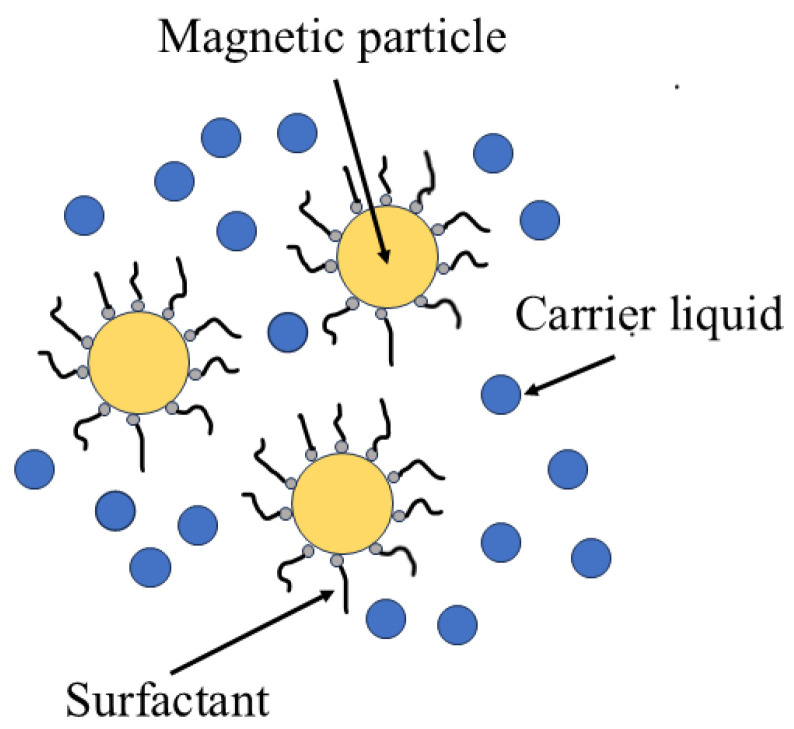
Composition and structural diagram of magnetic fluid.

In the late 20th century, Hoogerbrugge and Koelman first proposed a mesoscopic simulation technique called DPD for simulating complex fluid dynamics phenomena [21]. The popularity of the DPD model stems from its simple algorithm and wide applicability. Since the DPD method is a mesoscale numerical simulation technique, the solvent molecular clusters are considered dissipative particles. Therefore, in the case of magnetic fluids under consideration, there are two types of particles: magnetic particles and dissipative particles. The advantage of this method is that it enables access to longer time and length scales compared to what is achievable by conventional MD simulations. Figure 1 illustrates a schematic diagram of a typical Magnetic fluid.

### 2.1. Dissipative Particle Dynamics Models

We assume a system composed of N magnetic particles with mass m dispersed in a carrier liquid at thermodynamic equilibrium. In this study, the magnetic fluid is set as spherical particles, and we only need to consider the translational motion of the magnetic particles while neglecting their rotational motion.

There are three forces acting on a DPD particle: the conservative force $F_{ij}^{C}$ exerted by other particles, the dissipative force $F_{ij}^{D},$ which produces viscosity effects and drag forces among particles, and the random force $F_{ij}^{R}$ that generates thermal motion of particles. Taking these forces into account, the equation of motion for particle i can be expressed as follows [22]:

(1) $$m_{d}\frac{dv_{i}}{\;dt}=\sum_{j(\neq i)}\;F_{ij}^{C}+\sum_{j(\neq i)}\;F_{ij}^{D}+\sum_{j(\neq i)}\;F_{ij}^{R}$$

in which

(2) $$F_{ij}^{C}=\alpha w_{R}(r_{ij})e_{ij}$$

(3) $$F_{ij}^{R}=\sigma w_{R}(r_{ij})e_{ij}\zeta_{ij}$$

(4) $$F_{ij}^{D}=-\gamma w_{D}(r_{ij})(e_{ij}\cdot v_{ij})e_{ij}F_{ij}^{D}=-\gamma w_{D}(r_{ij})(e_{ij}\cdot v_{ij})e_{ij}$$

In the given equations, $m_{d}$ represents the mass of dissipative particle i, and $v_{i}$ represents its velocity. The subscripts indicate that, for example, $F_{ij}^{C}$ represents the force applied to particle i by particle *j*. Moreover, the constants α, σ, and γ denote the strength of the repulsive conservative force, random force, and dissipative force, respectively. The weight functions $w_{D}(r_{ij})$ and $w_{R}(r_{ij})$ are defined in such a way that inter-particle forces decrease as the distance between two particles increases. The expression used to describe $w_{R}(r_{ij})$ is as follows [23]:

(5) $$w_{R}(r_{ij})=\left\{\begin{array}{cc} 1-\frac{r_{ij}}{d_{c}} & \mathrm{for}\;r_{ij}\leq d_{c}\\ 0 & \mathrm{for}\;r_{ij}>d_{c} \end{array}\right.$$

The weight functions $w_{D}(r_{ij})$ and $w_{R}(r_{ij})$, as well as the constants γ and σ, must satisfy the following relationships, respectively [24]:

(6) $$w_{D}(r_{ij})=w_{R}^{2}(r_{ij})$$

(7) $$\sigma^{2}=2\gamma kT$$

In the equations provided above, $d_{c}$ represents the apparent diameter of dissipative particles. $r_{ij}$ denotes the relative position ($r_{ij}=\left|r_{ij}\right|$), $r_{ij}=r_{i}-r_{j}$; $e_{ij}$ is the unit vector indicating the direction from particle i to particle j, expressed as $e_{ij}=r_{ij}/r_{ij}$; $v_{ij}$ represents the relative, given by $v_{ij}=v_{i}-v_{j}$; k represents Boltzmann’s constant, and T denotes the liquid temperature. Additionally, $\zeta_{ij}$ is a random variable that induces the random motion of the particles.

By integrating Equation (1) with respect to time over a small time interval Δt from t to t+Δt, we can derive the finite difference equations that govern the particle motion in simulations.

(8) $$\Delta r_{i}=v_{i}\Delta t$$

(9) $$\begin{array}{rr} \Delta v_{i}= & \frac{\alpha }{m_{d}}\sum_{j(\neq i)}w_{R}(r_{ij})e_{ij}\Delta t-\frac{\gamma }{m_{d}}\sum_{j(\neq i)}w_{R}^{2}(r_{ij})(e_{ij}\cdot v_{ij})e_{ij}\Delta t\\ & +\frac{(2\gamma kT{)}^{1/2}}{m_{d}}\sum_{j(\neq i)}\;w_{R}(r_{ij})e_{ij}\theta_{ij}\sqrt{\Delta t} \end{array}$$

where $\theta_{ij}$ is the stochastic variable that must adhere to the following stochastic properties:

(10) $$\left\langle \theta_{ij}\right\rangle =0$$

(11) $$\left\langle \theta_{ij}\theta_{i^{\prime }j^{\prime }}\right\rangle =(\delta_{ii^{\prime }}\delta_{ij^{\prime }}+\delta_{ij^{\prime }}\delta_{ji^{\prime }})$$

In which $\delta_{ij}$ is the Kronecker delta. During the simulation, the stochastic variable $\theta_{ij}$ is sampled from a uniform or normal distribution with zero average value and unit variance.

### 2.2. Magnetic Particle Model

**Figure 2:**
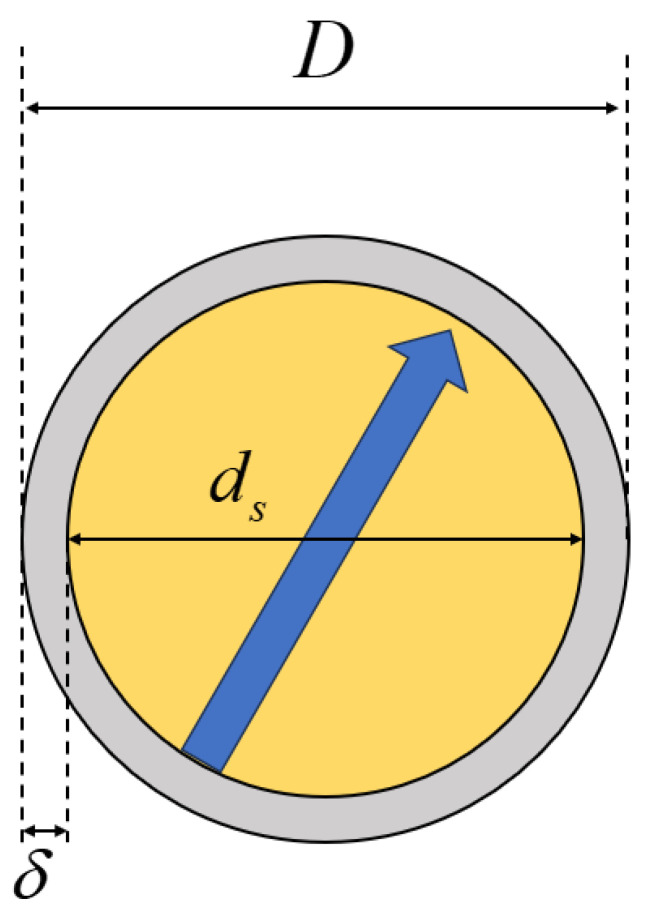
Model of surfactant-coated magnetic particles.

As shown in Figure 2, a magnetic particle is represented as a spherical particle with a central point dipole (positive and negative charges coincide at the same point) and is coated with a uniform steric layer, also known as a surfactant layer. In terms of notation, the diameter of the particle is denoted as $d_{s}$, the thickness of the steric layer as δ, and the overall diameter including the steric layer as $D=(d_{s}+2\delta )$.

The magnetic interaction energy between particles i and j, $u_{ij}^{\left(m\right)}$, the particle-field interaction energy, $u_{i}^{\left(H\right)}$, and the interaction energy resulting from the overlap of the steric layers, $u_{ij}^{\left(V\right)}$, are expressed as follows [22]:

(12) $$u_{ij}^{\left(m\right)}=\frac{\mu_{0}}{4\pi r_{ij}^{3}}\left\{m_{i}\cdot m_{j}-3(m_{i}\cdot t_{ij})(m_{j}\cdot t_{ij})\right\}$$

(13) $$u_{i}^{\left(H\right)}=-\mu_{0}m_{i}\cdot H$$

(14) $$u_{ij}^{\left(V\right)}=kT\lambda_{V}\left\{2-\frac{2r_{ij}/d_{s}}{t_{\delta }}\ln(\frac{D}{r_{ij}})-2\frac{r_{ij}/d_{s}-1}{t_{\delta }}\right\}$$

In this context, $\mu_{0}$ represents the permeability of free space, $m_{i}$ denotes the magnetic moment with $m_{0}=\left|m_{i}\right|$, and $t_{ij}$ is a unit vector defined as $r_{ij}/r_{ij}$, where $r_{ij}$ = $r_{i}-r_{j},\;r_{ij}=\left|r_{ij}\right|$. H denotes the applied magnetic field ($H=\left|H\right|$), and $t_{\delta }$ is defined as the ratio of the steric layer thickness δ to the radius of the solid portion of the particle, which can be expressed as $2\delta /d_{s}$. The nondimensional parameter $\lambda_{V}$, as observed in Equation (14), quantifies the strength of the steric particle–particle interaction relative to the thermal energy, represented by $\lambda_{V}=\pi d_{s}^{2}n_{s}/2$, where $n_{s}$ represents the number of surfactant molecules per unit area on the surface of the particle. The forces acting on particle i can be derived from Equations (12) and (14) [22]:

(15) $$F_{ij}^{\left(m\right)}=-\frac{3\mu_{0}}{4\pi r_{ij}^{4}}\left[-(m_{i}\cdot m_{j})t_{ij}+5(m_{i}\cdot t_{ij})(m_{j}\cdot t_{ij})t_{ij}-\left\{(m_{j}\cdot t_{ij})m_{i}+(m_{i}\cdot t_{ij})m_{j}\right\}\right]$$

(16) $$F_{ij}^{\left(V\right)}=\frac{kT\lambda_{V}}{\delta }\times \frac{r_{ij}}{r_{ij}}\ln(\frac{D}{r_{ij}}),\;(d_{s}\leq r_{ij}\leq D)$$

The movement of magnetic particles is described by Newton’s equations and is discretized in time to obtain finite difference equations that govern the particle motion in simulations.

(17) $$\Delta r_{i}=v_{i}\Delta t$$

(18) $$\Delta v_{i}=\sum_{j(\neq i)}F_{ij}\Delta t/m_{m}$$

In this equation, $m_{m}$ denotes the mass of the magnetic particles, and it is important to note that

(19) $$F_{ij}=F_{ij}^{\left(m\right)}+F_{ij}^{\left(V\right)}$$

**Figure 3:**
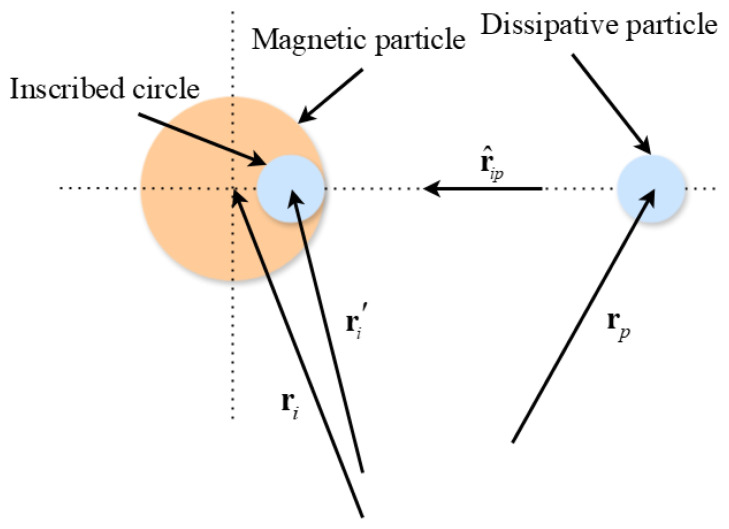
The interaction model between dissipative and magnetic particles.

In our approach, each colloidal particle is represented as a collective of dissipative particles. Normally, the interaction between a magnetic particle and the surrounding dissipative particles is considered to be the interaction between the surrounding dissipative particles and the constituent dissipative particles of the magnetic particle. However, in real dispersions, the nature of interactions between colloidal particles and solvent molecules depends on the specific characteristics of the dispersion. These interactions are heavily influenced by the mass-to-diameter ratio of colloidal particles relative to solvent molecules, as well as the properties of the interaction potential. As a result, instead of considering a colloidal particle solely as a group of dissipative particles, it may be feasible to employ a model potential to describe the interaction between magnetic and ambient dissipative particles. Figure 3 illustrates a schematic representation of the collision between a magnetic particle and a dissipative particle.

A suitable model for particle interaction that can be utilized is the Lennard-Jones model. The Lennard-Jones model is characterized by the following equation, which describes the interaction energy $u_{ip}$ between dissipative particle p and magnetic particle i [22]:

(20) $$u_{ip}=4n\varepsilon \left\{{\left(\frac{d_{c}}{{r_{ip}}^{\prime }}\right)}^{m}-{\left(\frac{d_{c}}{{r_{ip}}^{\prime }}\right)}^{n}\right\}$$

In this equation, ε represents a constant that quantifies the strength of the interaction, $r_{ip}^{\prime }=r_{i}^{\prime }-r_{p}$, $r_{ip}^{\prime }=\left|{r_{ip}}^{\prime }\right|$. The position vectors of the magnetic particle i and dissipative particle p are denoted as $r_{i}$ and $r_{p}$, respectively. In addition, $r_{i}^{\prime }$, the position vector representing the inscribed sphere is written as

(21) $$r_{i}^{\prime }=r_{i}-(D-d_{c}/2){\hat{r}}_{ip}$$

in which ${\overset{ˆ}{r}}_{ip}=r_{ip}/r_{ip}$, $r_{ip}=r_{i}-r_{p}$ and $r_{ip}=\left|r_{ip}\right|$. In the present simulation, the well-known Lennard-Jones potential is utilized, which is derived from the model potential by setting m to 12 and n to 6 in Equation (20). The force $F_{ip}^{(int)\;}$ exerted on dissipative particle p by magnetic particle i is determined by the interaction energy described in Equation (20), as follows:

(22) $$F_{ip}^{\left(\mathrm{int}\right)}=4n\varepsilon \left\{\frac{m}{n}{\left(\frac{d_{c}}{{r_{ip}}^{\prime }}\right)}^{m}-{\left(\frac{d_{c}}{{r_{ip}}^{\prime }}\right)}^{n}\right\}\frac{{\overset{ˆ}{r}}_{ip}}{{r_{ip}}^{\prime }}$$

The subsequent quantities are employed for the nondimensionalization of equations: d for distance, $m_{m}$ for mass, kT for energy, ${\left(kT/m_{m}\right)}^{1/2}$ for velocity, $d{\left(m_{m}/kT\right)}^{1/2}$ for time and kT/d for force. By using these quantities, we have [22]:

(23) $$\Delta r_{i}^{*}=v_{i}^{*}\Delta t^{*}$$

(24) $$\begin{array}{rr} \Delta v_{i}^{*}= & \frac{1}{m_{d}^{*}d_{c}^{*}}\alpha^{*}\sum_{j(\neq i)}w_{R}(r_{ij}^{*})e_{ij}\Delta t^{*}-\frac{1}{{\left(m_{d}^{*}\right)}^{1/2}d_{c}^{*}}\gamma^{*}\sum_{j(\neq i)}w_{R}^{2}(r_{ij}^{*})(e_{ij}\cdot v_{ij}^{*})e_{ij}\Delta t^{*}\\ & -\frac{1}{{\left(m_{d}^{*}\right)}^{3/4}d_{c}^{*1/2}}{\left(2\gamma^{*}\right)}^{1/2}\sum_{j(\neq i)}w_{R}(r_{ij}^{*})e_{ij}\theta_{ij}\sqrt{\Delta t^{*}}-\frac{1}{m_{d}^{*}}\sum_{k}F_{ki}^{(int)*}\Delta t^{*} \end{array}$$

where

(25) $$w_{R}(r_{ij}^{*})=\left\{\begin{array}{ll} 1-r_{ij}^{*}/d_{c}^{*} & \mathrm{for}\;r_{ij}^{*}/d_{c}^{*}\leq 1\\ 0 & \mathrm{for}\;r_{ij}^{*}/d_{c}^{*}>1 \end{array}\right.$$

(26) $$\alpha^{*}=\alpha \frac{d_{c}}{kT},\;\gamma^{*}=\gamma \frac{d_{c}}{{\left(m_{d}kT\right)}^{1/2}}$$

The number density of dissipative particles is nondimensionalized as

(27) $$n_{d}^{*}=n_{d}d^{2}=n_{d}d_{c}^{2}{\left(d/d_{c}\right)}^{2}={\hat{n}}_{d}^{*}/d_{c}^{*2}$$

In addition to $n_{d}^{*}$, the nondimensional density ${\hat{n}}_{d}^{*}$ based on the diameter of dissipative particles may be useful for quantifying the packing characteristics of the dissipative particles. The nondimensional number density of magnetic particles is expressed as $n_{m}^{*}=n_{m}d^{2}$. Similarly, the nondimensional form of correlative equation are expressed as:

(28) $$\Delta r_{i}^{*}=v_{i}^{*}\Delta t^{*}$$

(29) $$\Delta v_{i}^{*}=\sum_{j\neq i}F_{ij}^{*}\Delta t^{*}+\sum_{p}F_{ip}^{(int)*}\Delta t^{*}$$

(30) $$\begin{array}{rr} F_{ij}^{(m)*}= & -3\lambda \frac{1}{r_{ij}^{4*}}\left[-(n_{i}\cdot n_{j})t_{ij}+5(n_{i}\cdot t_{ij})(n_{j}\cdot t_{ij})t_{ij}-\left\{(n_{j}\cdot t_{ij})n_{i}\right.\right.\\ & \left.\left.+(n_{i}\cdot t_{ij})n_{j}\right\}\right] \end{array}$$

(31) $$F_{ij}^{(V)*}=\lambda_{V}\frac{1}{t_{\delta }^{*}}\times t_{ij}\ln(\frac{1}{r_{ij}^{*}})$$

in which $F_{ij}^{*}={F_{ij}}^{(m)*}+{F_{ij}}^{(V)*}$, $n_{i}$ is the unit vector denoting the direction of the magnetic moment $m_{i},$ expressed as $n_{i}=m_{i}/m_{0}(m_{0}=\left|m_{i}\right|).$ The nondimensional parameter λ in Equation (30) is the strength of magnetic particle interactions relative to the thermal energy, expressed as $\lambda =\mu_{0}m_{0}^{2}/4\pi D^{3}kT$. A parameter $\lambda_{s}={\left(D/d_{s}\right)}^{3}\;\lambda =(\mu_{0}{m_{0}}^{2}/4\pi {d_{s}}^{3}kT)$ is defined based on the diameter of the solid component. The expression of the force between a dissipative and a magnetic particle is written in nondimensional form as

(32) $$F_{ip}^{*}=\lambda_{\varepsilon }\left\{\frac{m}{n}{\left(\frac{d_{c}^{*}}{r_{ip}^{\prime *}}\right)}^{m}-{\left(\frac{d_{c}^{*}}{r_{ip}^{\prime *}}\right)}^{n}\right\}\frac{{\overset{ˆ}{r}}_{ip}}{r_{ip}^{\prime *}/d_{c}^{*}}$$

in which $\lambda_{\varepsilon }$ is a nondimensional parameter representing the strength of the interaction, expressed as $\lambda_{\varepsilon }=4n\varepsilon /(kTd_{c}^{*})$.

## 3. Modeling and Simulation of Magnetic Fluids

When the number of magnetic fluid particles is set to N, the area of this two-dimensional simulation box is represented by ${LL}^{2}$, $(LL{)}^{2}=\frac{N\pi }{4\varphi }\;$ when magnetic particle volume fraction φ is known. Unless explicitly stated otherwise, the designated duration for this task is 1,000,000 steps. The parameters utilized for the simulations conducted in this study are as follows: $\lambda_{s}=4$, $\lambda_{\varepsilon }=10$, $\gamma^{*}=10$, $\alpha^{*}=\gamma^{*}/10$, $d_{c}^{*}=0.4$, $m_{d}^{*}=0.01$, ${\hat{n}}_{d}^{*}=1$, $\Delta t^{*}=0.0001$, $\lambda_{V}=120$, volume fraction of magnetic particle φ is 0.2, the number of magnetic fluid particles is 100 and surfactant layer thickness $\delta^{*}=0.15$. The total number of simulation steps, $N_{\mathrm{timemx}\;}$, when the condition of $\Delta t^{*}N_{timemx\;}=100$ is met, it is anticipated to be adequate.

The time integration method is one of the key factors affecting the accuracy of simulation results in magnetic fluid simulations based on dissipative particle dynamics. Different time integration methods can have an impact on simulation results, so it is necessary to run and compare these methods under the same system parameters. The following are common time integration methods:

The Euler Scheme (ES) is one of the simplest integration methods, but it is still an effective time integration method in some cases. This method is based on obtaining the position and velocity of the next time step from the position and velocity of the previous time step. The expression is as follows:

(33) $$r_{i}(t+\Delta t)=r_{i}(t)+\Delta tv_{i}(t)$$

(34) $$v_{i}(t+\Delta t)=v(t)+\Delta t\frac{F_{i}(t)}{m_{i}}$$

(35) $$F_{i}(t+\Delta t)=F_{i}(r_{i}(t+\Delta t),v_{i}(t+\Delta t))$$

In the 1960s, the Verlet algorithm was proposed [25], in which an explicit expression for particle velocity appeared as follows:

(36) $$r_{i}(t+\Delta t)=r_{i}(t)+\Delta tv(t)+\frac{{\left(\Delta t\right)}^{2}F_{i}(t)}{2m_{i}}$$

(37) $$F_{i}(t+\Delta t)=F_{i}(r_{i}(t+\Delta t))$$

(38) $$v_{i}(t+\Delta t)=v_{i}(t)+\frac{\Delta t\left[F_{i}(t)+F_{i}(t+\Delta t)\right]}{2m_{i}}$$

**Figure 4:**
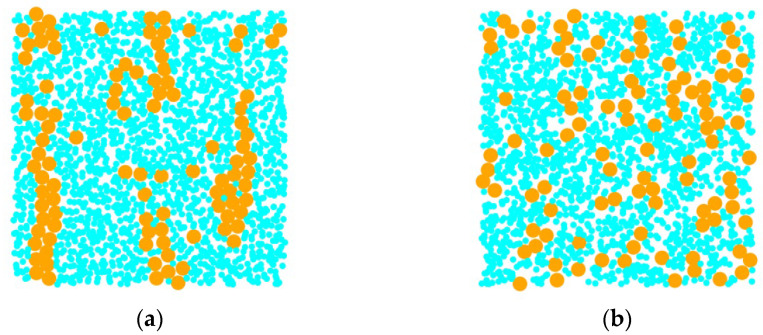
Microstructure of magnetic fluid obtained based on different time integration methods. (**a**) The results obtained by the ES algorithm; (**b**) the result obtained by Verlet algorithm.

Figure 4 shows the simulation results obtained using different algorithms. The small blue-green circles in the figure represent dissipative particles, while the large orange circles represent magnetic particles. It can be observed from the figure that, under the same conditions, there are a large number of magnetic particles gathering together to form coarse chain structures in the image obtained using the ES algorithm. However, at this time, the interaction between magnetic particles does not play an absolute dominant role relative to thermal energy, and under such conditions, large magnetic particle aggregation structures should not be formed.

The radial distribution function (RDF), *g*(*r*), defines the probability of two particles appearing at a given distance, *r*, and serves as an important tool for quantitatively characterizing the microstructure of Magnetic fluid. Specifically, for the two-dimensional radial distribution function, it is defined as follows [26]:

(39) $$g(q)=\frac{1}{N/A}\frac{s}{2\pi q△q}$$

In this context, A represents the area of the computational region containing N particles, and s represents the number of particles within a circular shell distance of q from the target particle, in the range of q to q *+* △q.

**Figure 5:**
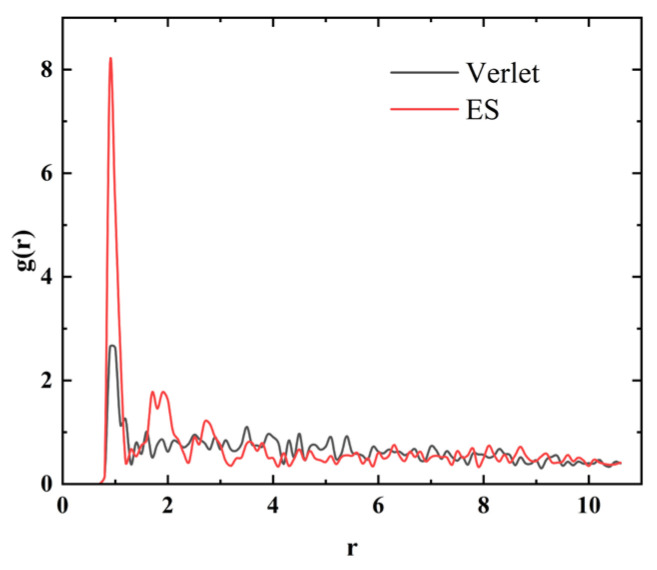
Radial distribution functions corresponding to different algorithms.

The radial distribution function corresponding to the ES algorithm has a higher peak near the integer radius *r*, indicating that compared to the Verlet algorithm, there are more magnetic particles distributed at these distances, corresponding to the chain like structure generated by the magnetic chain in Figure 5. The radial distribution function of the Verlet algorithm is relatively smooth, corresponding to the fact that most magnetic particles are still in an independent state.

**Figure 6:**
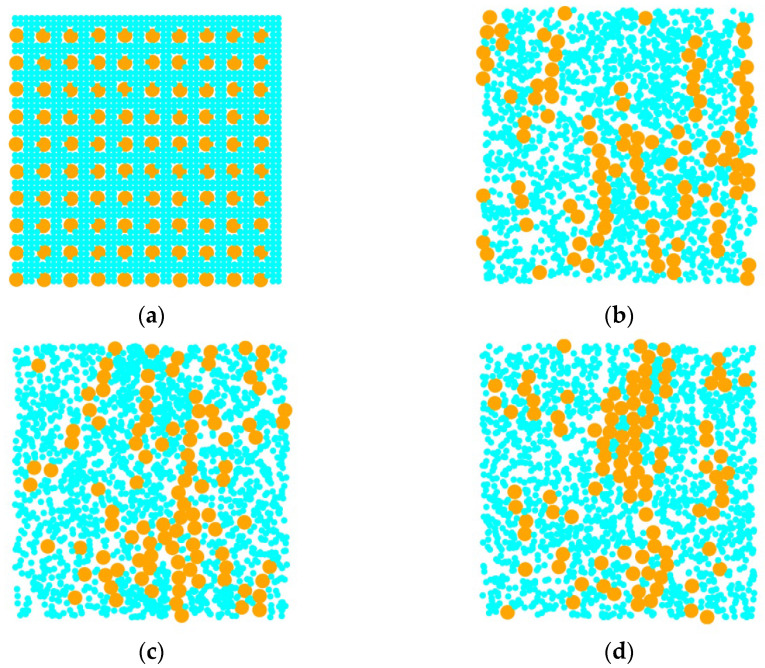
The dynamic process of the aggregation morphology of magnetic particles over time. (**a**) t*= 0; (**b**) t*= 10; (**c**) t*= 70; (**d**) t*= 100.

Figure 6 shows the dynamic evolution of magnetic particle structure over time. The small circles in the graph represent dissipative particles, while the large circles represent magnetic particles. From the figure, it can be seen that at the beginning of the simulation, the magnetic particles were uniformly distributed, and then dissipative particles were filled in the simulation area. With the increase in dimensionless time t*, the initially disordered distribution transforms into movement along the direction of the magnetic field, leading to aggregation and the emergence of long-chain structures. The aggregation structures of these ferromagnetic particles are in excellent agreement with the qualitatively obtained results from the experiments in [17].

## 4. Computational Findings and Analysis

### 4.1. Effect of the Molecular Mass of Carrier Liquid on the Structure of Magnetic Fluid

**Figure 7:**
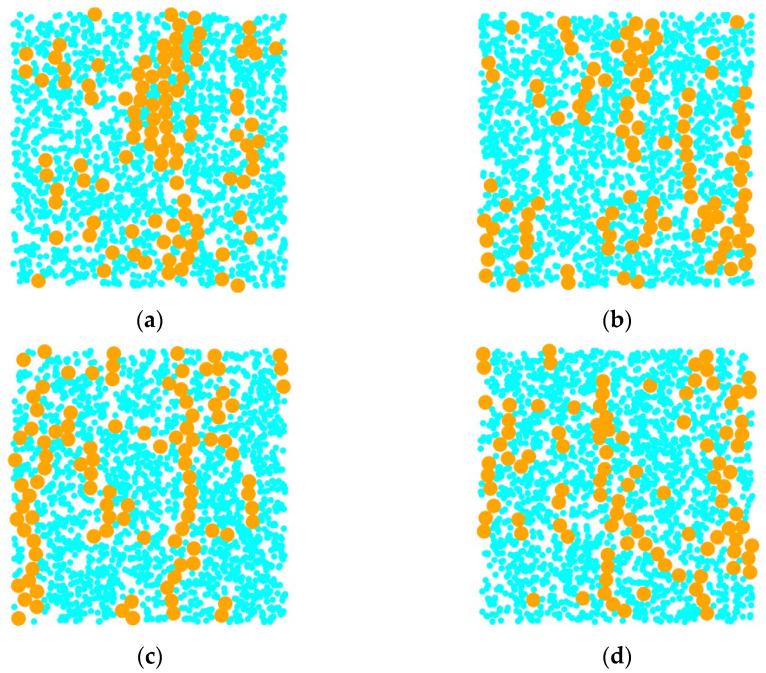
The structure of magnetic fluid changes with the mass of dissipative particles. (**a**) $m_{d}^{*}=0.01$; (**b**) $m_{d}^{*}=0.013$; (**c**) $m_{d}^{*}=0.015$; (**d**) $m_{d}^{*}=0.018$.

**Figure 8:**
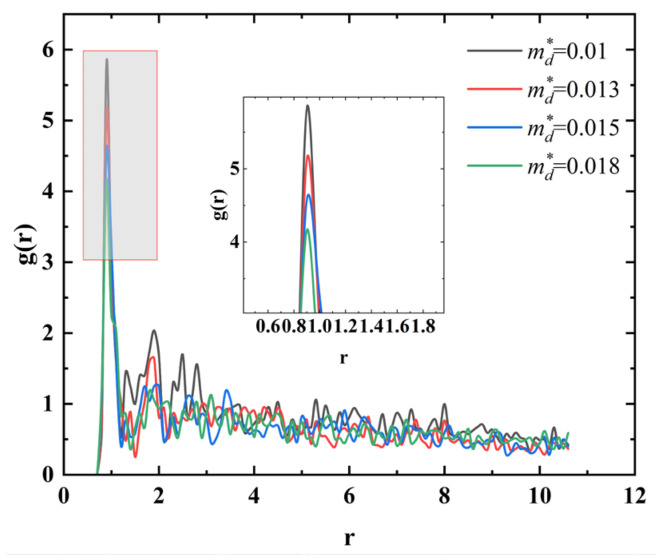
Radial distribution functions corresponding to different dissipative particle masses.

When the mass of dissipative particles is small, magnetic particles tend to form chain-like aggregation structures with larger radial sizes, as illustrated in Figure 7a. With an increase in the mass of dissipative particles (Figure 7b), the aggregation morphology of magnetic particles transitions into thinner chain-like structures. Further increasing the mass of dissipative particles leads to the formation of discrete cluster-like distributions, as shown in Figure 7c,d. These structural changes, analyzed through differential mass variations, indicate that the aggregate behavior of magnetic particles is highly sensitive to the mass of dissipative particles. As shown in Figure 8, with the increase in the mass of dissipative particles, the peak of the radial distribution function near *r* = 1 shows a decreasing trend, indicating the gradual dissociation of cluster structures among magnetic particles. This phenomenon suggests a corresponding increase in the proportion of independent magnetic particles in the system. Moreover, the results demonstrate that changes in the weight of the carrier liquid directly influence the motion behavior of magnetic particles in a magnetic field, thereby regulating the morphology of chain-like structures in magnetic fluids.

### 4.2. Effect of the Magnetic Particle Interaction Strength on the Structure of Magnetic Fluids

In a strong magnetic field environment, the interaction strength between magnetic particles plays a crucial role in regulating their structure. With the increase in interaction strength, the interaction between magnetic particles intensifies, leading to a stronger tendency for magnetic particles to form arranged chain-like structures. This evolutionary process of structure is essential for a profound understanding of the behavior and properties of magnetic particles in a strong magnetic field.

**Figure 9:**
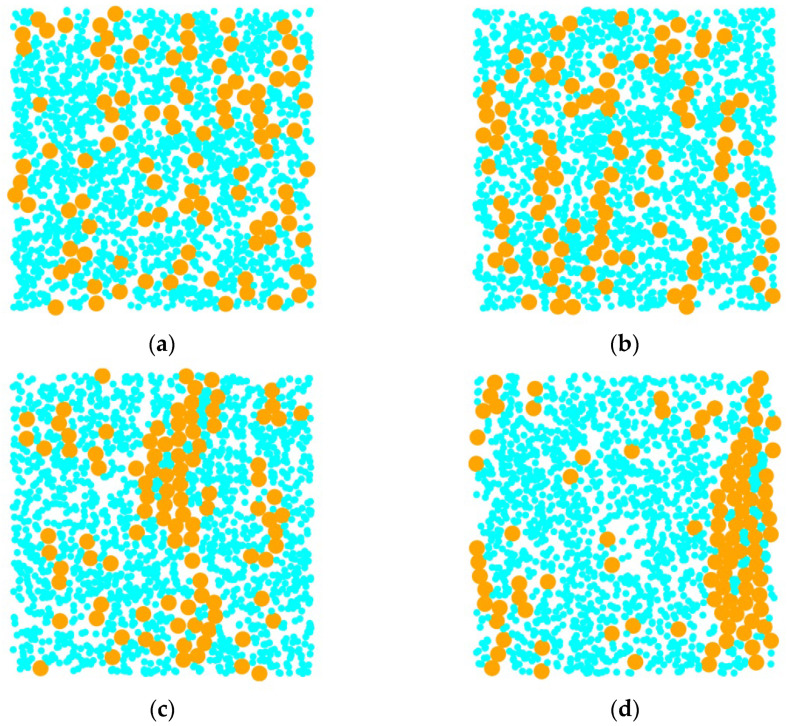
Influence of magnetic particle interaction strength variations on magnetic fluid structure. (**a**) $\lambda_{s}\;$= 4; (**b**) $\lambda_{s}\;$= 6; (**c**) $\lambda_{s}\;$= 8; (**d**) $\lambda_{s}\;$= 10.

**Figure 10:**
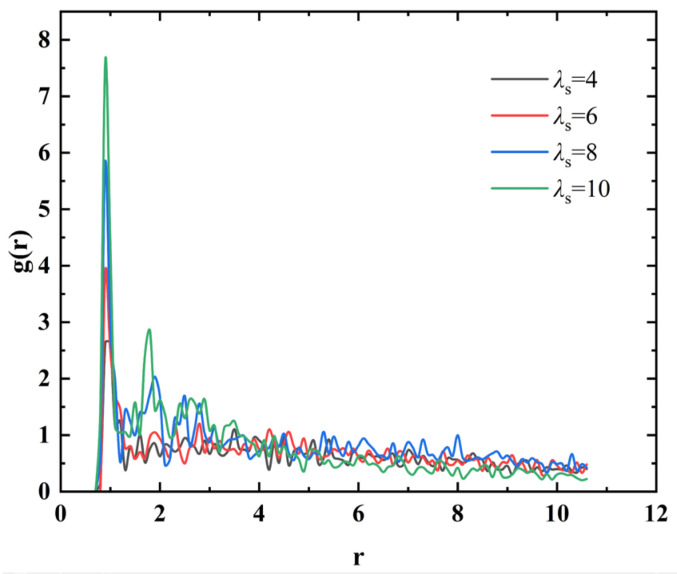
Impact of particle–particle magnetic interactions on the RDF *g*(*r*) of magnetic particles.

As shown in Figure 9a, when the interaction strength between magnetic particles is low ($\lambda_{s}=4$), although some particles come into contact with each other to form cluster structures, the structure is relatively loose, indicating a significant influence of Brownian motion. At the same time, there are a large number of particles in a free state without interacting with other magnetic particles. With an increase in $\lambda_{s}$, as shown in Figure 9b,c, the cluster structure gradually transitions to a chain-like structure, while the number of free particles begins to decrease. As shown in Figure 9d, when the interaction strength between magnetic particles increases to a higher value, its influence on the structure of magnetic fluids relative to thermal energy takes precedence. The majority of magnetic particles form chain-like structures and are ordered along the direction of the magnetic field. This structural transition process is clearly reflected in the radial distribution function: as shown in Figure 10, with the increase of $\lambda_{s}$, the peak intensity at *r* = 1 continues to strengthen, reflecting the structural evolution of particles from an independent state to chain-like aggregation.

## 5. Conclusions

This study employs DPD simulations to investigate the structural evolution of magnetic fluids under external magnetic fields, revealing key factors influencing their tunable microstructures. The computational model, incorporating magnetic nanoparticles and solvent particles, demonstrates qualitative agreement with the established literature, confirming the validity of the chosen simulation approach. Through radial distribution function analysis, we highlight the critical roles of the mass of solvent molecules and magnetic interaction strength in governing the fluid’s chain-like nanoparticle organization. These findings advance the understanding of magnetic fluid behavior and provide a foundation for optimizing their design in practical applications. Future work could explore additional environmental conditions and extend to 3D models to further refine predictive modeling.

## Data Availability

Data will be made available on request.

## References

[1] Zhao Y., Wu D., Lv R.-Q., Ying Y. (2014). Tunable characteristics and mechanism analysis of the magnetic fluid refractive index with applied magnetic field. IEEE Trans. Magn..

[2] Suriyanto, Ng E.Y.K., Kumar S.D. (2017). Physical mechanism and modeling of heat generation and transfer in magnetic fluid hyperthermia through Néelian and Brownian relaxation: A review. Biomed. Eng. Online.

[3] Ren B., Song X., Zhao L., Jin Y., Bai S., Cui C., Wang J. (2022). Water-based Fe_3_O_4_ magnetic fluid-coated *Aspergillus niger* spores for treating liquid contaminated with Cr(VI). Environ. Res..

[4] Hamzah S., Ying L.Y., Azmi A.A.A.R., Razali N.A., Hairom N.H.H., Mohamad N.A., Harun M.H.C. (2021). Synthesis, characterisation and evaluation on the performance of ferrofluid for microplastic removal from synthetic and actual wastewater. J. Environ. Chem. Eng..

[5] Yang X., Yang Q., Yang W., Guo B., Chen L. (2018). Analysis of Adjustable Magnetic Fluid Damper in DC Magnetic Field for Spacecraft Applications. IEEE Trans. Appl. Supercond..

[6] Li X., Fan X., Li Z., Zhu M. (2023). Failure mechanism of magnetic fluid seal for sealing liquids. Tribol. Int..

[7] Munshi M.M., Patel A.R., Deheri G.M. (2019). Lubrication of rough short bearing on shliomis model by ferrofluid considering viscosity variation effect. Int. J. Math. Eng. Manag. Sci..

[8] Wang D., Yi Z., Ma G., Dai B., Yang J., Zhang J., Yu Y., Liu C., Wu X., Bian Q. (2022). Two-channel photonic crystal fiber based on surface plasmon resonance for magnetic field and temperature dual-parameter sensing. Phys. Chem. Chem. Phys..

[9] Yan L., Wang Q., Yin B., Xiao S., Li H., Wang M., Liu X., Wu S. (2023). Research on Simultaneous Measurement of Magnetic Field and Temperature Based on Petaloid Photonic Crystal Fiber Sensor. Sensors.

[10] Yu Q., Li X.-G., Zhou X., Chen N., Wang S., Li F., Lv R.-Q., Nguyen L.V., Warren-Smith S.C., Zhao Y. (2022). Temperature compensated magnetic field sensor using magnetic fluid filled exposed core microstructure fiber. IEEE Trans. Instrum. Meas..

[11] Abdullah H., Mitu S.A., Ahmed K. (2020). Magnetic fluid-injected ring-core-based micro-structured optical fiber for temperature sensing in broad wavelength spectrum. J. Electron. Mater..

[12] Farzinpour M., Toghraie D., Mehmandoust B., Aghadavoudi F., Karimipour A. (2020). Molecular dynamics study of barrier effects on Ferro-nanofluid flow in the presence of constant and time-dependent external magnetic fields. J. Mol. Liq..

[13] Satoh A., Chantrell R.W., Kamiyama S.-I., Coverdale G.N. (1996). Two-dimensional Monte Carlo simulations to capture thick chainlike clusters of ferromagnetic particles in colloidal dispersions. J. Colloid Interface Sci..

[14] Hao L., Xinhua L., Yongzhi L. (2011). The lattice Boltzmann simulation of magnetic fluid. Procedia Eng..

[15] Zhao Z., Torres-Díaz I., Vélez C., Arnold D., Rinaldi C. (2016). Brownian dynamics simulations of magnetic nanoparticles captured in strong magnetic field gradients. J. Phys. Chem. C.

[16] Sun Y., Wei Z., Zhou J., Mao A., Bian D. (2024). Modification of magnetorheological fluid and its compatibility with metal skeleton: Insights from multi-body dissipative particle dynamics simulations and experimental study. Phys. Fluids.

[17] Lv R.-Q., Zhao Y., Xu N., Li H. (2013). Research on the microstructure and transmission characteristics of magnetic fluids film based on the Monte Carlo method. J. Magn. Magn. Mater..

[18] Li W., Ouyang J., Zhuang X. (2016). Dissipative particle dynamics simulation for the microstructures of ferromagnetic fluids. Soft Mater..

[19] Moghadam M.G.E., Shahmardan M.M., Norouzi M. (2019). Dissipative particle dynamics modeling of a mini-MR damper focus on magnetic fluid. J. Mol. Liq..

[20] Xu Z.-D., Sun C.-L. (2021). Single-double chains micromechanical model and experimental verification of MR fluids with MWCNTs/GO composites coated ferromagnetic particles. J. Intell. Mater. Syst. Struct..

[21] Hoogerbrugge P.J., Koelman J.M.V.A. (1992). Simulating microscopic hydrodynamic phenomena with dissipative particle dynamics. Europhys. Lett. (EPL).

[22] Satoh A. (2010). Introduction to Practice of Molecular Simulation: Molecular Dynamics, Monte Carlo, Brownian Dynamics, Lattice Boltzmann and Dissipative Particle Dynamics.

[23] Satoh A., Chantrell R.W. (2006). Application of the dissipative particle dynamics method to magnetic colloidal dispersions. Mol. Phys..

[24] Espanol P., Warren P. (1995). Statistical mechanics of dissipative particle dynamics. Europhys. Lett..

[25] Verlet L. (1967). Computer “experiments” on classical fluids. I. Thermodynamical properties of lennard-jones molecules. Phys. Rev..

[26] Phan-Thien N., Mai-Duy N., Khoo B.C. (2014). A spring model for suspended particles in dissipative particle dynamics. J. Rheol..

